# Cluster-Locating Algorithm Based on Deep Learning for Silicon Pixel Sensors

**DOI:** 10.3390/s23094383

**Published:** 2023-04-28

**Authors:** Fatai Mai, Haibo Yang, Dong Wang, Gang Chen, Ruxin Gao, Xurong Chen, Chengxin Zhao

**Affiliations:** 1 University of Chinese Academy of Sciences, Beijing 100049, China; maifatai20@mails.ucas.ac.cn (F.M.); yanghaibo@impcas.ac.cn (H.Y.); chengang@impcas.ac.cn (G.C.); xchen@impcas.ac.cn (X.C.); 2 Institute of Modern Physics, Chinese Academy of Sciences, Lanzhou 730000, China; 3 Advanced Energy Science and Technology Guangdong Laboratory, Huizhou 516003, China; 4 PLAC, Key Laboratory of Quark & Lepton Physics (MOE), Central China Normal University, Wuhan 430079, China; dongwang@mail.ccnu.edu.cn; 5 School of Electronic Engineering, Heilongjiang University, Harbin 150006, China; 2211828@s.hlju.edu.cn

**Keywords:** particle physics, deep learning, cluster locating, YOLO, RCNN, transformer

## Abstract

The application of silicon pixel sensors provides an excellent signal-to-noise ratio, spatial resolution, and readout speed in particle physics experiments. Therefore, high-performance cluster-locating technology is highly required in CMOS-sensor-based systems to compress the data volume and improve the accuracy and speed of particle detection. Object detection techniques using deep learning technology demonstrate significant potential for achieving high-performance particle cluster location. In this study, we constructed and compared the performance of one-stage detection algorithms with the representative YOLO (You Only Look Once) framework and two-stage detection algorithms with an RCNN (region-based convolutional neural network). In addition, we also compared transformer-based backbones and CNN-based backbones. The dataset was obtained from a heavy-ion test on a Topmetal-M silicon pixel sensor at HIRFL. Heavy-ion tests were performed on the Topmetal-M silicon pixel sensor to establish the dataset for training and validation. In general, we achieved state-of-the-art results: 68.0% AP (average precision) at a speed of 10.04 FPS (Frames Per Second) on Tesla V100. In addition, the detection efficiency is on the same level as that of the traditional Selective Search approach, but the speed is higher.

## 1. Introduction

As a leading platform for heavy-ion scientific research in China and one of a few large-scale full-ion accelerating systems in the world, the Heavy Ion Research Facility in Lanzhou (HIRFL) [1] and the High Intensity Heavy-ion Accelerator Facility (HIAF) [2] have put forward higher requirements for detector technology. The development of the Monolithic Active Pixel Sensor (MAPS) [3,4] provides an unprecedented signal-to-noise ratio, energy resolution, spatial resolution, and readout speed in particle physics experiments. The MAPS integrates the sensing node and readout circuit into one chip. It collects the charge deposited in particles passing through the sensors and then measures the positions of particle hits. In addition, some newly designed MAPSs [5,6] also record the energy and timing information of the particle hits. The typical MAPS-based detectors at HIRFL and HIAF are vertex and tracking detectors in Electron-ion colliders in China (EicC) [7] and the beam-positioning system at physics terminals [8]. Besides these benefits, adopting MAPS technology increases the data volume. Therefore, high-performance cluster-locating technology is highly required in MAPS-based systems. Energy deposited by particle hits is collected by pixels in the MAPS, and these pixels form a connected region, which is called a cluster. Cluster-locating technology finds the clusters induced by particle hits in a given event, the aim of which is to compress the data volume and improve the accuracy and speed of particle detection.

Cluster-locating algorithms have already been widely used in physics experiments, such as at the European Organization for Nuclear Research (CERN) [9,10,11,12] and the Compressed Baryonic Matter (CBM) experiments [13]. In particle physics experiments, charged particles are produced during beam collisions. These charged particles interact with the material around them as they pass through the vertex and tracking detectors, causing them to deposit energy that can be detected and measured. The track and energy information are critical for studying the properties of these particles. A cluster-locating algorithm helps identify the hit positions of charged particles in each detector layer along their track with high efficiency and accuracy. This contributes to the trajectory and energy information reconstruction of the particles. In addition, an online cluster-locating algorithm will also benefit the data compression since only data that contain clusters need to be stored.

The current commonly used cluster-locating algorithms mainly belong to the following categories:

1. In regional detection algorithms [14], samples (images) are divided into partitions as a prerequisite for consistency judgment. Each partition contains the maximum number of consistent pixels. For example, if we want to locate the area of a cluster, we start from the seed pixel in the cluster, which has the highest energy, then look for fired pixels in the seed pixel’s neighborhood, and take the fired pixel as the new seed pixel. This domain expansion process is repeated until no more fired pixels exist. The key to this method is to set reasonable guidelines for domain expansion.
2. Edge detection methods, such as Canny [15], the Sobel operator, etc., aim to find items’ edges. The performance of edge detection algorithms highly depends on the quality of the edges’ features. For example, if we want to use edge detection methods to locate clusters, we must obtain the edge information of the clusters. However, the edges of the clusters may be blurred, which cannot guarantee the continuity and closure of the edges. In this case, the detection accuracy can be relatively poor. Thus, the edge detection method has weak robustness for cluster location.
3. A clustering algorithm [16] is an unsupervised machine learning algorithm. For example, when we want to locate clusters, a clustering algorithm will divide each data frame into two dissecting subsets: the background and the foreground (clusters in our case). Taking the value of each pixel as the input, certain distance measurement methods (Euclidean distance, Manhattan distance, etc.) calculate the similarity between the data in each subset. The above process is continuously and iteratively optimized so that the data in the same subset are as similar to each other as possible, and the data in different subsets are as different as possible. Finally, we obtain two sets: the pixels of the objects (clusters) and the background pixels. Clustering algorithms are easy to deploy and execute. However, they are usually sensitive to isolated pixels and do not utilize the spatial information provided by the pixels in the samples. As a result, clustering algorithms highly rely on the features’ quality.

As one of the most famous scientific research trends, deep learning techniques are widely applied in many fields, such as computer vision, natural language processing, and speech recognition. Object detection techniques using deep learning technology have been actively studied [17,18]. They demonstrate significant potential to overcome the limitations of cluster-locating algorithms, such as those mentioned above.

Object detection algorithms with deep learning include two-stage and one-stage detection models. The two-stage model generates a series of candidate boxes, then extracts the features of these candidate boxes, and finally classifies and regresses the target. In contrast, the one-stage detection algorithm directly classifies and regresses the target. The RCNN series [17,18,19,20] is an essential representative of the two-stage detection model, which presents high accuracy in target location and recognition. On the other hand, the YOLO series [21,22,23,24,25] is a relatively popular one-stage detection model, which has a good balance between speed and accuracy.

Since the advent of AlexNet [26], convolutional neural networks (ConvNets) have been the dominant model architecture for computer vision. Since then, ConvNets have become a widely researched topic. Several more effective and more scalable convolutional neural networks have been proposed, such as VGGNet [27], GoogLeNet [28], ResNe(X)t [29,30], DenseNet [31], MobileNet [32], and EfficientNet [33]. ConvNets have been widely used as backbone networks to improve performance in visual tasks. Since a ConvNet has inductive bias and translation invariance, it is a good design principle for object detection.

On the other hand, self-attention [34] has been introduced as a recent advance. Unlike the convolution operation, the key to self-attention is to produce a weighted average of values computed from hidden units. Therefore, the interactions between the input signals of self-attention are determined by themselves rather than by their relative positions, such as convolution, which allows self-attention to capture long-range interactions. With the success of Transformer [34] based on the self-attention mechanism in NLP (natural language processing), many previous studies have also tried to introduce the self-attention mechanism into computer vision, and Transformer has also become the mainstream network architecture. Recently, Vision Transformer (ViT) [35] with only vanilla Transformer layers achieved good performance on ImageNet-1K [36]. In addition, ViT pre-training on the large-scale weakly labeled JFT-300M dataset [37] can obtain comparable results to state-of-the-art (SOTA) ConvNets. Swin Transformer [38] was the first to show that Transformer can be used as a generic visual backbone and achieve SOTA performance in a range of vision tasks.

## 2. Method

**Figure 1:**
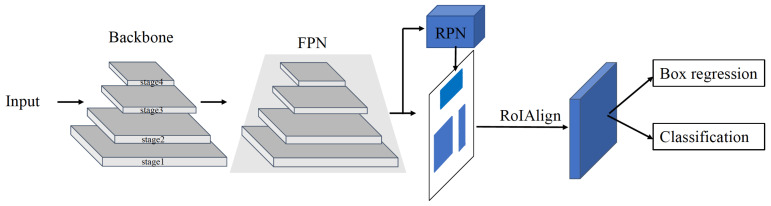
Two-stage model.

**Figure 2:**
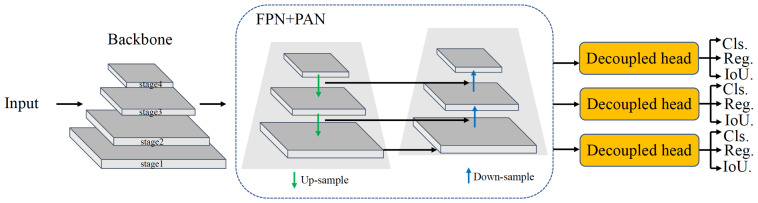
One-stage model.

To apply a deep learning approach in cluster-locating algorithms for CMOS pixel sensors in physics experiments, we performed the following research:

- We performed a beam test on the Topmetal-M [6] silicon pixel sensor at the External Target Facility at the Heavy Ion Research Facility in Lanzhou. In this test, we recorded the cluster data induced by energy of 320 MeV/u, with an average beam intensity of several thousand counts/cm${}^{2}$/s. After pre-processing, we used the images that contained the cluster data to form the dataset for training and verification.
- We constructed both one- and two-stage detection algorithms, as shown in Figure 1 and Figure 2. We use Transformer-based and CNN-based backbones for feature extraction at different stages in the one- and two-stage detection algorithms. Additionally, each model comes in four different sizes. The two-stage detection algorithms first generate region proposals from images and then generate the final object boxes from the region proposals, while the one-stage object detection algorithms do not need the region proposal stage and directly generate the object’s class probability and position coordinate values.

### 2.1. The Backbone Network and Its Variants

The backbone network is an essential feature extractor for a object detection task. It takes the image as the input and outputs the feature maps of the corresponding input image. However, most of the backbone networks used for object detection come from a network of classification tasks, taking out the last fully connected layer of the classification tasks. Therefore, a complex backbone network is needed to meet the requirements of high precision and a more accurate application. This study used two backbone networks: the Swin Transformer backbone based on the Transformer and the ConvNeXt backbone based on the convolutional neural network.

In addition, to continuously improve the model’s ability, different sizes of backbone variants were used, namely, Swin-T/S/B/L and ConvNeXt-T/S/B/L. In essence, the model is constantly deepened and widened. As shown in Table 1, ConvNeXt variants differ in the number of channels and blocks in each stage. As summarized in Table 2, the Swin Transformer variants differ in the channel number in hidden layers, the number of layers, and the number of heads in multi-head attention at each stage. The number of channels and heads doubles at each new stage. ConvNeXt and Swin Transformer have the same number of channels for variants of the same size type at each stage, such as Swin-T and ConvNeXt-T.

### 2.2. The Two-Stage Model

As shown in Figure 1, the two-stage model performs feature extraction on the input image through the neural network with a feature pyramid network (FPN) [39]. The backbone extracts different scales of region-of-interest (RoI) features from different stages of the feature pyramid. The FPN contains a bottom-up path and a top-down path with a lateral connection. The path from the bottom to the top builds a hierarchical structure, which can extract features at different scales. The path from the top to the bottom produces higher-resolution features through upsampling, and its semantic information is stronger. Each lateral connection combines feature maps of the same scale in the top-down and bottom-up paths to form a new pyramid level, and each level makes independent predictions. Higher-resolution feature maps help detect small targets, while lower-resolution feature maps contain rich semantic information, so constructing the FPN is essential.

The Region Proposal Network (RPN) [17] has classification and regression branches. The classification branch divides the anchor boxes into positive and negative anchors through the softmax function. The regression branch calculates the offset of the boundary box regression of the anchor box to obtain a more accurate candidate box. Region proposals with a wide range of scales and aspect ratios are generated with the RPN. The classification of region proposals is completed simultaneously, which divides the candidates into two categories: background and target. The RPN accelerates the generating speed of candidate object bounding boxes because it shares a common set of convolution layers with the detection network. In addition, a new method for detecting objects of different sizes uses multi-scale anchors as references. The anchors greatly simplify the generation of region proposals of various sizes without needing multiple scales of images or features. The anchors have three aspect ratios. This method parameterizes the candidate bounding box relative to the reference anchor box, optimizes the prediction box’s position by measuring the distance between it and its corresponding ground-truth box, and processes the bounding box, including closing the excess part of the positive anchor beyond the image boundary to the edge of the image, removing the tiny positive anchor, and carrying out non-maximum suppression (NMS) of the remaining positive anchors. The RPN only makes a preliminary prediction of the object’s location to be detected.

Then, we use RoIAlign [20] to extract a small feature map from each proposal. RoIAlign converts the area corresponding to the position coordinates of the region proposals into a fixed-sized feature, which is convenient for subsequent classification and boundary box regression operations. RoI pooling uses two quantization steps, resulting in misalignments between the input and output pixels of the network. To solve the misalignment problem, RoIAlign does not use quantization operations and keeps the floating-point coordinates of each proposal in the feature map. First, the bilinear interpolation method calculates the exact value of the four regularly sampled proposals’ feature map positions. Then, max or average pooling is used to aggregate the results to obtain the fixed-length feature of each proposal. RoIAlign can improve the accuracy of small-target detection because it uses floating-point numbers. By aligning with the small target of the original image, it can be restored to the original position with greater accuracy. This aspect is well adapted to our particle cluster detection task.

Finally, the network head for bounding-box recognition (classification and box regression) is applied to each proposal. The aim of classification here is to identify which category all previous positive anchors belong to, which differs from the only two-category classification in the RPN. Then, bounding box regression is performed on the proposals to obtain a more accurate final predicted box.

In this paper, the backbone variants we use are Swin Transformer based on Transformer architectures with attention mechanisms and ConvNeXt [40] based on convolutional neural network (CNN) architectures.

### 2.3. The One-Stage Model

As shown in Figure 2, the one-stage model generally comprises three parts: a backbone, a neck, and a head. The backbone mainly determines the feature representation ability. Meanwhile, its design significantly impacts the inference efficiency since it carries a high computation cost. The backbone is divided into multiple stages to extract features at different scales and facilitates features fusion later. Usually, the neck, located between the backbone and the head network, further improves the diversity and robustness of the features. The neck aggregates low-level physical features with high-level semantic features and then builds up pyramid feature maps at all levels. A neck is composed of several bottom-up paths and top-down paths. Building a pyramid on the feature map better solves the problems induced by changes in the scales of the targets. The famous examples are the feature pyramid network (FPN) [39] and the Path Aggregation Network (PAN) [41]. The FPN improves the size of the feature map to solve the problem of the scale sizes of different targets in detection. The PAN increases the network’s depth, improves the network’s robustness, and enhances the target-positioning ability to a certain extent. The FPN can capture strong semantic features from top to bottom, and the PAN conveys strong positioning characteristics from bottom to top. Hence, combining these two modules can effectively realize target positioning. The head consists of several convolutional layers, which use 1×1 convolution to adjust the number of channels on the three scales of the neck network. It predicts the final detection results according to multi-level features assembled by the neck. The decoupled head for classification and localization, widely used in object detection [42,43], speeds up the convergence.

The network architecture of our one-stage model comprises the following modules:

1. The backbone for feature extraction over an entire image is Swin Transformer and ConvNeXt. Swin Transformer constructs hierarchical feature maps, achieves linear computation complexity by computing self-attention locally within non-overlapping windows, and introduces shifted windows for cross-window connections to enhance the modeling power. ConvNeXt adopts a hierarchical structure and uses a larger kernel size in convolution to increase the receptive field.
2. The neck used to aggregate feature maps from different stages consists of an FPN and PAN, which enhance the entire feature hierarchy with the accurate localization of signals in lower layers by bottom-up path augmentation. To correspond to the output of three different scales, the FPN is constructed based on the feature map from backbone stages 2∼4.
3. The head used to predict the classes and bounding boxes of objects is YOLOX [44], which is divided into the classification, regression, and IoU branches.

## 3. Dataset Establishment

### 3.1. Cluster Data Generation in Heavy-Ion Campaigns

**Figure 3:**
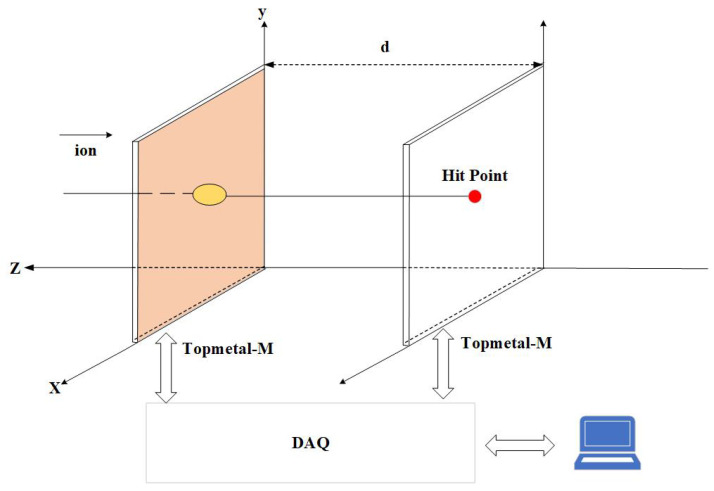
Data acquisition system.

We performed a heavy-ion beam campaign to establish a dataset for training and verifying the algorithms. Beam tests were performed at the External Target Facility [45] at the Heavy Ion Research Facility in Lanzhou (HIRFL). As shown in Figure 3, the test setup consists of two Topmetal-M [6] silicon pixel sensors placed in parallel and the data acquisition (DAQ) system.

Topmetal-M [6] has the function of a Monolithic Active Pixel Sensor (MAPS). This sensor is implemented with a new 130 nm high-resistivity (>1 kΩ· cm) CMOS process. It has a size of 18 mm × 23 mm, and each pixel cell has dimensions of 40 μm × 40 μm. The peripheral circuit was placed at the left and bottom of the pixel matrix. The pixel matrix is divided into 16 sub-arrays, which can be read simultaneously, and the maximum clock rate is 40 MHz.

The energy of the ${}^{40}$Ar beam emitted from the terminal was 320 MeV/u, and the average beam intensity was thousands of counts/cm${}^{2}$/s. The Ar particles hit and pass through the first Topmetal-M sensor and the second Topmetal-M sensor consecutively. When an Ar particle traverses the depletion region [46] of the Topmetal-M sensor, it will lose some of its energy to produce electron–hole pairs. The charge collection diode in each pixel collects the electrons, and the in-pixel circuit converts the electron into an analog signal. These pixels form a cluster. The DAQ system controls and reads data from the two chips and then transfers the data to a computer. A detailed description of this experiment is presented in [8].

### 3.2. Cluster Data Pre-Processing

The cluster data captured in the heavy-ion experiment were then processed through the following steps to build the dataset.

Data pre-processing: The collected data are parsed and divided into many groups; the dimensions of each group are H×W×F, where *H* and *W* represent the row and column numbers of the pixel array in the Topmetal-M sensor, and *F* is the number of frames.

We pre-process the data through background acquisition, background suppression, and data filtering to extract the effective particle clusters.

Background acquisition: To extract the particle cluster more accurately, we calculate the background of each H×W×F group of data as follows:

1. For *F* frames, the mean $m_{ij}$ and variance $\sigma_{ij}$ of each position $p_{ij}\;i\in \left[1,H\right],j\in \left[1,W\right]$ are determined; i,j represents the *i*-th row, *j*-th column.
2. According to the 3σ criterion, the value at each position $p_{ij}$ of the *F* frame data is retained at a value of $\left[m_{ij}-3\sigma_{ij},m_{ij}+3\sigma_{ij}\right]$, and values exceeding $\left[m_{ij}-3\sigma_{ij},m_{ij}+3\sigma_{ij}\right]$ are eliminated.
3. The values reserved at each position are averaged to obtain the value $m_{ij}$’ of each position, which is used as background information and denoted as b.

Background suppression: We subtract the corresponding background b from each data group to suppress the background.

Data filtering: To retain effective clusters, a single cluster’s energy *E* and size *S* are used as thresholds.

**Figure 4:**
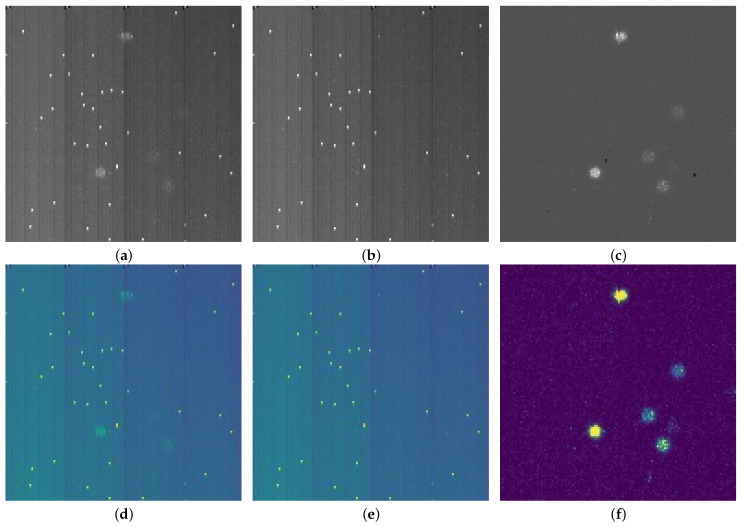
Data pre-processing: (**a**) source image 1, (**b**) background image 1, (**c**) result of data pre-processing, (**d**) pseudo-color image of source, (**e**) pseudo-color image of background, (**f**) pseudo-color image of pre-processing result.

As shown in Figure 4, the upper row is a gray scale of the pre-processed images. The lower row is the corresponding pseudo-color image, where the visual effect of data pre-processing is more straightforward. This pre-processing mechanism is also part of the cluster-locating algorithms used when implementing the deep learning approach in the physics experiments. It can remove empty frames and invalid clusters, thus improving the speed and accuracy of cluster detection.

**Figure 5:**
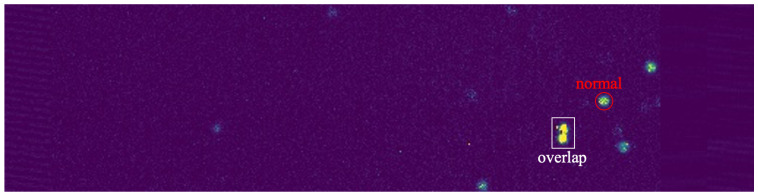
The shape type of the clusters: the red circle represent a single normal cluster, and the white rectangle indicates overlapping clusters.

**Figure 6:**
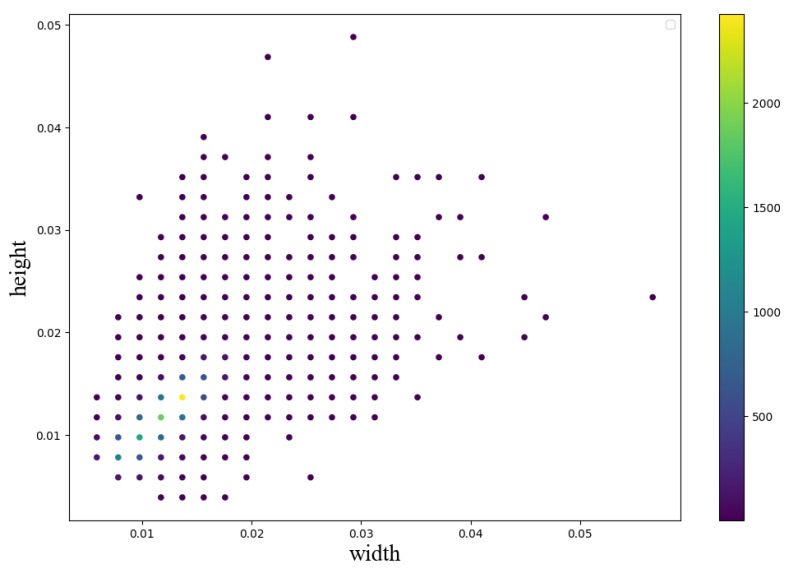
The height and width distribution of clusters: both height and width are ratios relative to the width of the original image.

The pre-processing data establish the dataset, where the ratio of the training set to the validation set is 3:1. As shown in Figure 5, the clusters generally have two types: the first one is a single cluster (red circle), and the other is several overlapping clusters (white rectangle). A single cluster presents a circle-like shape, and the occupancy of single clusters is higher than that of overlapping clusters. Figure 6 shows the height and width distribution of the clusters. The overall relationship between the height and width of the clusters is linear, which conforms to the shape type of the cluster.

## 4. Experimental Results

### 4.1. Implementation Details and Performance

We validated and tested the one-stage model (Section 2.3) and two-stage model (Section 2.2) with Tesla V100 [47]. There are two main metrics for detection, namely, AP and FPS, where AP is the approximate value of the area under the P-R (precision–recall) curve. Precision and recall are calculated as follows:

(1) $$\begin{array}{cc} P & =\frac{TP}{TP+FP} \end{array}$$

(2) $$\begin{array}{cc} R & =\frac{TP}{TP+FN} \end{array}$$

where *TP* (true positive) represents the number of Intersection-over-Union (IoU) [48] values of the prediction box and the GT (ground truth) box greater than the threshold thres (the general value is 0.5), *FP* (false positive) represents the number of IoU values of the prediction box and the GT box less than the threshold thres, and *FN* (false negative) indicates that the number of GT boxes has not been detected. In our experiment, AP is expressed as the average value with an IoU threshold of 50∼95% (with a step of 5%). AP${}_{50}$ is the AP value with an IoU threshold of 50%, with an analogous definition for AP${}_{75}$.

The one-stage model: We used stochastic gradient descent (SGD) for training with an initial lr=0.001. The weight decay was 0.0005, and the SGD momentum was set to 0.9. A warm-up with five epochs was also utilized. We adopted Mosaic [24,25], MixUp [49], RandomAffine, and RandomFlip for data augmentation.

**Figure 7:**
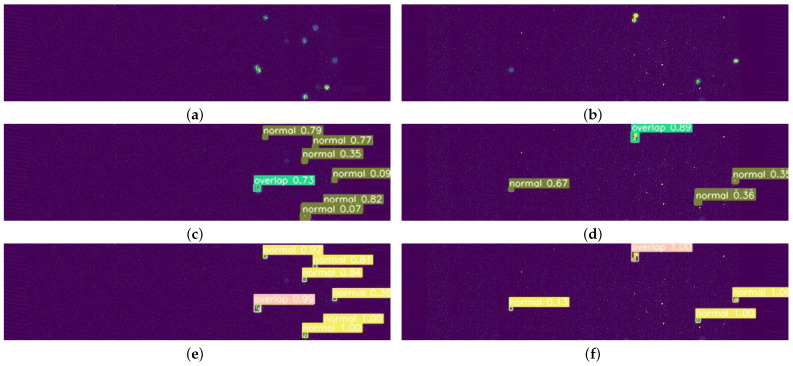
Test results: (**a**) image 1, (**b**) image 2, (**c**) results of image 1 in one-stage model (Swin-T), (**d**) results of image 2 in one-stage model (Swin-T), (**e**) results of image 1 in two-stage model (ConvNeXt-L), (**f**) results of image 2 in two-stage model (ConvNeXt-L).

Table 3 demonstrates the performance results obtained with the one-stage model. The model’s parameters, with Swin Transformer and ConvNeXt as backbones, are on the same scale for each set of variants. In addition, the consumption of computation power is also on the same scale. For example, Swin-L has 246.56 M parameters and consumes 69.52 FLOPs, and ConvNeXt-L has 247.80 M parameters and 57.67 FLOPS. In general, the detection algorithms with Swin Transformer as the backbone achieve significantly higher accuracy than those with ConvNeXt as the backbone but have a slower speed. The test results of Swin-T are shown in Figure 7c,d, in which both classification and localization have high accuracy. Swin-T obtains the best result of 57.2% AP in terms of accuracy, which is an 8.0% higher AP compared to that of ConvNeXt-T at the same scale of the model. However, ConvNeXt-T obtains the fastest speed.

The two-stage model: We employed an AdamW [50] optimizer with a cosine decay learning rate scheduler and 500 epochs of linear warm-up. An initial learning rate of 0.0001, a weight decay of 0.05, and a momentum of 0.9 were used. For data augmentation, we adopted RandomFlip and multi-scale.

Table 4 shows the results of the two-stage model. The models with ConvNeXt as the backbone and Swin Transformer as the backbone have similar sizes; for example, Swin-B has 104.03 M parameters and ConvNeXt-B has 104.86 M parameters. In general, the detection algorithm with ConvNeXt as the backbone is better in accuracy and speed than that with Swin Transformer as the backbone. ConvNeXt-L achieves the best detection accuracy of 68.0% AP, which is a 1.3% higher AP than that of Swin-L. ConvNeXt-T reaches the fastest speed at 27.24 FPS, 2.71 times faster than ConvNeXt-L. Figure 7e,f show the test results of ConvNeXt-L, which achieves better performance on classification and localization.

### 4.2. Comparison between Object Detection Algorithms

**Figure 8:**
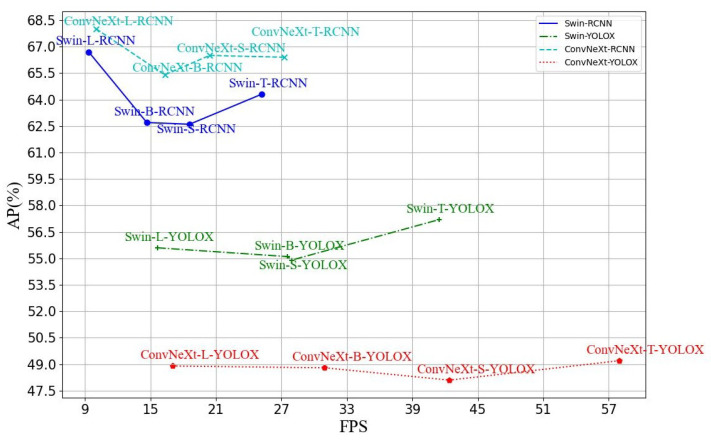
Comparison of the speed and accuracy of different object detection algorithms.

Figure 8 summarizes the performance of all object detection algorithms used in this study. The two-stage detection algorithms show a lower speed than the one-stage detection algorithms. For example, ConvNeXt-T’s speed has dropped by 30.70 FPS from the one-stage model to the two-stage model.

However, the one-stage detection algorithms show significantly lower accuracy than the two-stage detection algorithms; the possible reasons are as follows: (1) As illustrated in Figure 5 and Figure 6, the size of the targets to be detected is relatively tiny (many are less than 4% of the original image), and the number of small targets in a single frame is significant. In addition, (2) the background of the target to be detected is complex, and the noise interference is substantial. (3) The number of targets is unbalanced, resulting in an unbalanced sample. All of the above reasons can make it challenging to achieve high accuracy with one-stage detection. Therefore, all object detection algorithms use the FPN to make detecting small targets and targets with large changes in scale as accurate as possible.

Furthermore, Swin Transformer and ConvNeXt show different results in one-stage and two-stage detection algorithms. In the one-stage detection algorithms, Swin Transformer outperforms ConvNeXt in accuracy, and the opposite is the case for speed. On the other hand, in two-stage detection algorithms, ConvNeXt surpasses Swin Transformer in precision and speed.

Therefore, we can choose different detection algorithms to achieve the proper combination of speed, accuracy, model size, and computational resource consumption for different applications depending upon requirements. In our experiments, we used ConvNext-L-RCNN to accomplish the cluster detection tasks since it shows the best performance in accuracy and an acceptable speed.

### 4.3. Detection Efficiency and Fake Rate

When applying the cluster-locating algorithm to physics experiments, the performance we concern ourselves with is the efficiency of locating clusters and the rate of introducing fake clusters. This paper defines detection efficiency as the ratio between the number of detected true clusters and the total number of true clusters and defines the fake rate as the ratio between the number of fake clusters and the total detected clusters. As the first step, we must exclude the fake clusters induced by the sensor’s noise. Thus, we took 200 frames of data generated without particle hits for this study. Table 5 shows that no clusters were detected when no particles hit the sensor. Then, we used 200 frames of data generated with particle hits to study the detection efficiency and the fake rate. The total number of clusters in these 200 frames is 660. We used the Selective Search (SS) [51] approach as the reference for the deep learning approaches. Table 6 shows the performance comparison results. The detection algorithms (Swin-T-YOLOX, ConvNeXt-T-YOLOX, and ConvNeXt-L-RCNN) based on deep learning have nearly the same level of detection efficiency compared to the Selective Search approach. As for detection speed, [17] indicates that SS has a speed of 0.5FPS, which is nearly an order of magnitude slower than that of Faster RCNN [17]. The one-stage algorithms (Swin-T-YOLOX and ConvNeXt-T-YOLOX) have slightly higher efficiency than the two-stage algorithms (ConvNeXt-L-RCNN and ConvNeXt-T-RCNN), but their performance in the fake rate is an obvious drawback. The results also align with the results in Figure 8, where the one-stage algorithms show faster speed and the two-stage algorithms present better accuracy. Therefore, the one-stage algorithms can be responsible for locating preliminary clusters during online data compression. On the other hand, the two-stage algorithms can perform high-precision cluster detection tasks.

## 5. Conclusions

To compress a large amount of online data and improve accuracy and speed in extracting clusters induced by particle hits at HIRLF and HIAF, we studied the performance of a deep learning approach applied to cluster-locating algorithms. We constructed one-stage and two-stage detection algorithms, with Swin Transformer and ConvNext as the backbones. Heavy-ion tests were performed on the Topmetal-M silicon pixel sensor to establish a dataset for training and validation. In general, the two-stage detection algorithms demonstrate significantly better accuracy in object localization and recognition, and the weakness in speed is acceptable for the current applications at HIRFL and HIAF. For example, the two-stage detection algorithm (ConvNeXt-L-RCNN) demonstrates the best detection accuracy of 68.0% AP, while the one-stage detection algorithm (ConvNeXt-T-YOLOX) achieves the fastest speed of 57.94 FPS. The deep-learning-based cluster-locating algorithm presents nearly the same detection efficiency as the traditional Selective Search approach while having a speed one order higher. Furthermore, a study on the fake rate shows that the one-stage detection algorithms show great potential for online data compression, and the two-stage detection algorithms can perform high-precision cluster detection tasks. The research in this paper aims at providing practical experience for applying cluster-locating algorithms in physics experiments. In the future, we expect to improve the detection algorithm to reach a good balance between speed and accuracy.

## Figures and Tables

**Table 1:** ConvNeXt variants.

Backbone	Channels	Blocks
ConvNeXt-T	[96, 192, 384, 768]	[3, 3, 9, 3]
ConvNeXt-S	[96, 192, 384, 768]	[3, 3, 27, 3]
ConvNeXt-B	[128, 256, 512, 1025]	[3, 3, 9, 3]
ConvNeXt-L	[192, 384, 768, 1536]	[3, 3, 9, 3]

**Table 2:** Swin Transformer variants.

Backbone	Channels	Layers	Heads
Swin-T	[96, 192, 384, 768]	[2, 2, 6, 2]	[3, 6, 12, 24]
Swin-S	[96, 192, 384, 768]	[2, 2, 18, 2]	[3, 6, 12, 24]
Swin-B	[128, 256, 512, 1025]	[2, 2, 18, 2]	[4, 8, 16, 32]
Swin-L	[192, 384, 768, 1536]	[2, 2, 18, 2]	[6, 12, 24, 48]

**Table 3:** Using different variants of Swin Transformer and ConvNeXt as the backbone and YOLOX as the head for one-stage model training results: comparison of Swin Transformer and ConvNeXt as the backbone in terms of AP (%) (red font).

Models	AP(%)	AP${}_{50}\left(\%\right)$	AP${}_{75}\left(\%\right)$	FPS	Params.(M)	FLOPs(G)
ConvNeXt-T-YOLOX	49.2	85.0	54.0	57.94	38.44	8.64
Swin-T-YOLOX	57.2 (**+8.0**)	90.0	66.7	41.47	38.12	9.93
ConvNeXt-S-YOLOX	48.1	84.0	50.4	42.40	62.34	14.55
Swin-S-YOLOX	54.9 (**+6.8**)	87.2	64.1	27.93	61.71	17.42
ConvNeXt-B-YOLOX	48.8	83.9	53.6	30.96	110.48	25.74
Swin-B-YOLOX	55.1 (**+6.3**)	87.7	64.9	27.57	109.65	30.93
ConvNeXt-L-YOLOX	48.9	84.4	51.0	17.04	247.80	57.67
Swin-L-YOLOX	55.6 (**+6.7**)	87.1	65.5	15.64	246.56	69.52

**Table 4:** Using different variants of Swin Transformer and ConvNeXt as the backbone and an RCNN as the head for two-stage model training results: comparison of Swin Transformer and ConvNeXt as the backbone in terms of AP (%) (red font) and comparison of one-stage and two-stage models in terms of FPS (black bold).

Models	AP(%)	AP${}_{50}$(%)	AP${}_{75}$(%)	FPS	Params.(M)	FLOPs(G)
Swin-T-RCNN	64.3	90.8	82.6	25.18(**−16.29**)	44.72	27.65
ConvNeXt-T-RCNN	66.4 (**+2.1**)	91.9	83.9	27.24 (**−30.70**)	45.03	26.36
Swin-S-RCNN	62.6	89.4	80.1	18.58(**−9.35**)	66.02	34.76
ConvNeXt-S-RCNN	66.5 (**+3.9**)	93.3	82.3	20.41 (**−21.99**)	66.64	31.88
Swin-B-RCNN	62.7	90.1	81.1	14.66(**−12.91**)	104.03	45.85
ConvNeXt-B-RCNN	65.4 (**+2.7**)	90.3	82.0	16.31 (**−14.65**)	104.86	40.65
Swin-L-RCNN	66.7	92.8	84.8	9.34(**−6.30**)	212.50	77.46
ConvNeXt-L-RCNN	68.0 (**+1.3**)	93.4	86.2	10.04 (**−7.00**)	213.74	65.61

**Table 5:** Fake clusters induced by sensor noise.

Methods	Fake Clusters
SS	0
Swin-T-YOLOX	0
ConvNeXt-T-YOLOX	0
ConvNeXt-L-RCNN	0
ConvNeXt-T-RCNN	0

**Table 6:** Comparison of detection efficiency and fake rate. The superscript 1 and 2 are detection efficiency and fake rate formula respectively.

Methods	Detected True Clusters	Detected Clusters	Detection Efficiency (%)	Fake Rate (%)
SS	650	672	98.49	3.23
Swin-T-YOLOX	648	811	98.18	20.10
ConvNeXt-T-YOLOX	646	863	97.88	25.14
ConvNeXt-L-RCNN	641	641	97.12	0
ConvNeXt-T-RCNN	629	629	95.30	0

^1^ $\mathrm{detection}\;\mathrm{efficiency}=\frac{\mathrm{detected}\;\mathrm{true}\;\mathrm{clusters}\;}{\mathrm{all}\;\mathrm{true}\;\mathrm{clusters}}$ ^2^ $\mathrm{fake}\;\mathrm{rate}=\frac{\mathrm{detected}\;\mathrm{clusters}-\mathrm{detected}\;\mathrm{true}\;\mathrm{clusters}\;}{\mathrm{detected}\;\mathrm{clusters}}$.

## Data Availability

Not applicable.
